# Comparative Molecular and Antimicrobial Analysis of *Lactococcus garvieae* and *Lactococcus petauri* from Marine and Freshwater Fish Farms in the Mediterranean

**DOI:** 10.3390/ani16020277

**Published:** 2026-01-16

**Authors:** Daniel González-Martín, María Ubieto, Silvia del Caso, Elena Planas, Imanol Ruiz-Zarzuela, Celia Sanz, José Luis Arnal

**Affiliations:** 1 Exopol S.L., Polígono Río Gállego D/14, 50840 Zaragoza, Spain; 2 Biofarm West Med, Biomar Iberia S.A., Carretera A-62, Km. 99, Apdo 16, 34210 Palencia, Spain; 3 Laboratory of Fish Pathology, Facultad de Veterinaria, Instituto Agroalimentario de Aragón-IA2, Universidad de Zaragoza, C/Miguel Servet 177, 50013 Zaragoza, Spain

**Keywords:** antimicrobial susceptibility, aquaculture, European seabass, gilthead seabream, *Lactococcus garvieae*, *Lactococcus petauri*, molecular diagnostics, One Health, piscine lactococcosis, rainbow trout

## Abstract

Piscine lactococcosis is an emerging bacterial disease that threatens freshwater and marine aquaculture in the Mediterranean region. This study characterized isolates of *Lactococcus garvieae* and *Lactococcus petauri* from farmed fish through molecular identification, genomic typing and antimicrobial susceptibility testing. A total of 39 bacterial strains were analyzed using species-specific real-time PCR assays, multilocus sequence typing and broth microdilution to determine minimum inhibitory concentrations. Results suggest a temporal shift in freshwater systems, where *L. garvieae* predominated in earlier isolates (mainly ST13, CC4), while *L. petauri* (ST14, CC14) appears as the dominant species in recent years. In marine fish, only *L. garvieae* was detected, mainly ST95 (CC95), a lineage previously reported in Europe. Molecular variability was found in both species with lineages capable of infecting livestock and humans. Amoxicillin displayed promising results; florfenicol showed moderate activity, while flumequine exhibited no inhibitory effect. Oxytetracycline and trimethoprim–sulfamethoxazole showed variable results requiring prudent use. These region-specific susceptibility profiles provide updated baseline data to guide empirical antimicrobial therapy while awaiting laboratory confirmation, highlighting the evolution of lactococcosis in aquaculture and emphasizing the need for molecular surveillance, antimicrobial stewardship, and vaccine updates within a One Health framework to mitigate impacts on Mediterranean aquaculture and public health.

## Simple Summary

Fish farming in the Mediterranean is a major industry increasingly threatened by lactococcosis, a bacterial disease that causes substantial production losses and poses a potential zoonotic risk. We analyzed 39 bacterial isolates from farmed fish to determine the species involved, elucidate transmission pathways, compare intra-species strain diversity, and assess antimicrobial susceptibility to identify optimal therapeutic options. We used molecular assays to identify bacterial species and track clonal lineages, and we determined minimum inhibitory concentrations (MICs) for key antimicrobials. In freshwater trout farms, earlier isolates were predominantly *Lactococcus garvieae*, whereas in recent years *Lactococcus petauri* has become prevalent. In marine farms, only *Lactococcus garvieae* was detected, predominantly the same emergent lineage reported in marine European production and distinct from the freshwater lineages described to date. Across isolates, amoxicillin exhibited low MICs (suggesting high susceptibility), florfenicol showed intermediate activity, and flumequine was ineffective at the concentrations tested. Oxytetracycline and trimethoprim–sulfamethoxazole yielded variable results requiring prudent use. These region-specific, updated susceptibility profiles provide veterinarians with actionable baseline data to guide empirical antimicrobial therapy decisions while awaiting confirmatory laboratory results, supporting improved diagnostics, prudent stewardship, enhanced animal welfare, reduced farm losses, and public health protection.

## 1. Introduction

Aquaculture is a key contributor to global food production and human nutrition. While capture fisheries have remained stable since the 1980s, aquaculture continues to expand, and in 2022 global aquaculture fish production exceeded that of capture fisheries [1]. This growth underscores both the importance of the sector and its vulnerability to emerging infectious diseases. The expansion of aquaculture involves higher stocking densities, which increase host contact rates and favor pathogen transmission [2]. At the same time, the geographic expansion and movement of live aquatic animals connect previously isolated production systems. Together, these factors facilitate pathogen spread and enhance the establishment of emerging infectious diseases [3].

In Europe, aquaculture finfish production reached 524,373 metric tons in 2023, encompassing both freshwater (continental) and marine farming systems [4]. Among freshwater species, rainbow trout (*Oncorhynchus mykiss*)—traditionally the species most affected by lactococcosis [5]—was predominant, with a production of 414,908 tons and an estimated value of EUR 2387 thousand. Common carp (*Cyprinus carpio*) followed with 150,997 tons and a value of EUR 505 thousand [4]. Marine aquaculture was dominated by European seabass (*Dicentrarchus labrax*), accounting for 86,744 tons and EUR 623 thousand, and gilthead seabream (*Sparus aurata*), with 108,090 tons and EUR 588 thousand [4].

Beyond Europe, neighboring Mediterranean countries such as Turkey, Egypt, Tunisia, and Iran also sustain high aquaculture production, largely based on the same key species, including European seabass, gilthead seabream, mullet, and trout [1]. The overlap in farmed species, combined with intensive trans-Mediterranean trade and shared aquatic ecosystems, creates strong epidemiological connectivity across the region. As a result, the Mediterranean basin can be considered a single epidemiological unit, in which emerging infectious diseases may spread across national boundaries, posing significant biosecurity challenges.

Piscine lactococcosis is a significant septicemic and zoonotic disease with a global distribution, causing high mortality rates in both marine and freshwater aquaculture species. Disease-associated mortalities have been reported across the Americas, Africa, Europe, Asia and Oceania [5]. Among the most severely affected species, rainbow trout [6] and Japanese amberjack (*Seriola quinqueradiata*) [7] stand out due to their economic relevance and susceptibility. Clinically, lactococcosis is characterized by acute to subacute hemorrhagic septicemia, often associated with high mortality during warm seasons when water temperatures exceed 16 °C [5]. Affected fish typically exhibit anorexia, lethargy, abnormal swimming, exophthalmia (unilateral or bilateral), skin darkening, and petechial hemorrhages [5,8]. Internal lesions include widespread vascular damage, hepatosplenomegaly, pericarditis, and hemorrhages in visceral organs and the brain [5,8].

The first case of piscine lactococcosis reported in Europe occurred in rainbow trout in Spain in 1988 [9]. Since then, the disease has been detected in numerous European countries, including, among others, the United Kingdom [10], Portugal [11], France [12], Italy and Greece [13], affecting freshwater aquaculture farms of rainbow trout. Alarmingly, more recently, marine farms of European seabass [14] and gilthead seabream [15] along the Italian coast have also been affected. This positions piscine lactococcosis as an emerging disease in the Mediterranean Sea. Importantly, this sea is characterized by intense movements of both wild and farmed fish [16,17,18]. Movement of live aquaculture animals is frequent among European countries (both for freshwater and marine species). For instance, countries such as Greece, Italy, France and Spain export and import juveniles and broodstock regularly [19]. Given the broad host range of *Lactococcus garvieae* and the presence of susceptible hosts in the Mediterranean, the pathogen poses a significant risk to marine aquaculture species produced there.

Outbreaks have been associated with mortalities exceeding 50% in rainbow trout, together with reduced growth and diminished marketability [5,12]. Lactococcosis also causes substantial economic losses due to increased labor, treatment, and mortality costs. In Italy, outbreaks in rainbow trout farms account for 9–28% of total turnover [6]. Beyond the economic burden, lactococcosis-causing bacteria are recognized as potential zoonotic agents associated with fish handling and raw fish consumption [20,21].

Piscine lactococcosis is a bacterial disease historically attributed to *L. garvieae*, formerly classified as *Streptococcus garvieae* and later as *Enterococcus seriolicida* before its current taxonomic status was established [5,22,23,24]. In 2017, *Lactococcus petauri* was described from a sugar glider sample [25], and retrospective studies revealed that most freshwater outbreaks in Europe were actually caused by this species [13]. A third closely related species, *Lactococcus formosensis*, originally isolated from fermented broccoli [26], has since been linked to fish infections and may have been previously misidentified as *L. garvieae* [27]. These three taxa, *L. garvieae*, *L. petauri*, and *L. formosensis*, are now recognized as “lactococcosis-causing bacteria” (LCB). Although *L. formosensis* has mainly been reported in Asia [28] and the Americas [29,30,31], its recent detection in rainbow trout from Europe [32] suggests a wider distribution and underscores ongoing diagnostic challenges due to their clinical indistinguishability [13,33].

Routine culture with commonly used biochemical and phenotypic identification methods cannot reliably differentiate these species, nor can more advanced platforms such as matrix-assisted laser desorption/ionization time-of-flight mass spectrometry (MALDI-TOF MS). Therefore, molecular techniques based on real-time PCR (qPCR) have been developed to enable fast, sensitive and specific diagnosis [34,35,36]. Additionally, genetic characterization by sequencing multiple genes with the technique multilocus sequence typing (MLST) [37] has been used in different countries to carry out epidemiological studies [38]. This tool provides valuable insights for establishing epidemiological relationships in different hosts and ecological contexts.

At the farm level, outbreaks are difficult to control, representing a major concern since this disease leads to high mortality rates in high-value fish species. Previous studies have reported that both *L. garvieae* and *L. petauri* exhibit resistance to antibiotics widely used in aquaculture [8,39,40], highlighting the need to conduct antibiotic sensitivity tests prior to implementing therapeutic treatments. The in vitro technique of determining the minimum inhibitory concentration (MIC) by broth microdilution is currently considered the gold standard for antimicrobial susceptibility testing [41,42,43].

The regulation of antibiotic treatments in aquaculture is complex, as different countries apply distinct authorization frameworks despite sharing, in a broad sense, a common epidemiological context. In Spain, only a few antibiotics are authorized for bacterial infections in aquaculture, and their use is limited to certain fish species. Oxytetracycline remains the only drug approved for treating *L. garvieae* infections in several species, including eels, carp, seabream, seabass, turbot, and salmonids [44,45]. Florfenicol and flumequine are licensed in Spain exclusively for the treatment of *Aeromonas salmonicida* infections in trout [46,47]. In Italy, however, a different regulatory approach is applied, and the sulfadiazine–trimethoprim combination is licensed for the treatment of vibriosis, yersiniosis, and *aeromoniasis* [48]. Moreover, although no amoxicillin formulations are currently approved for fish, its growing off-label use underscores the need for authorized products specifically developed for aquaculture [49].

Beyond the need to control infections caused by these bacteria in fish, it is important to consider the One Health approach, as *Lactococcus* spp. isolates have also been reported in non-aquatic species such as cows, pigs, cats and dogs [50]. Furthermore, these pathogens have also been found in food products such as meat and milk [51]. Human infections have also been documented, either as opportunistic agents or associated with clinical conditions such as urinary tract infections and cholecystitis [51,52,53].

This study aimed to characterize *Lactococcus* spp. isolates obtained from Mediterranean aquaculture species using molecular detection, genomic typing by MLST and antimicrobial susceptibility testing using MIC by broth microdilution.

## 2. Materials and Methods

### 2.1. Bacterial Strain Collection

A total of 39 bacterial isolates classified as *Lactococcus* spp. were used in this study. Each isolate corresponded to an individual clinical case and was obtained from diseased fish sampled at aquaculture farms located in Mediterranean countries. Ten isolates were provided by the Fish Pathology Laboratory at the University of Zaragoza. They were collected between 2006 and 2014 and had previously been identified as *Lactococcus* sp. The remaining 29 strains were isolated from the material submitted to the laboratory Exopol (Zaragoza, Spain) for diagnostic testing between 2021 and 2024. Several submissions were provided under client confidentiality and/or lacked complete provenance metadata; therefore, precise farm locations and country-level information cannot be disclosed. For each isolate, we report the year of isolation and the host/environment category (freshwater vs. marine with host species) together with the year of isolation. Samples submitted to the diagnostic laboratory were stored under refrigeration (4 °C) during transport and processed within 24–48 h of arrival. Long-term storage of isolates was performed at −80 °C in a bacterial freezing medium containing milk and glycerol.

Seven isolates from 2012 to 2013 were non-viable at the time of this study, likely due to prolonged storage under suboptimal conditions, and were therefore used only for molecular analyses.

Of the total, 20 strains came from marine host species and 16 from freshwater species, and for 3 the origin was not specified (Table 1). Among the 20 marine isolates, the host species were European seabass (*Dicentrarchus labrax*) (*n* = 15), gilthead seabream (*Sparus aurata*) (*n* = 3) and unspecified (*n* = 2). Freshwater isolates were exclusively obtained from rainbow trout (*Oncorhynchus mykiss*) (*n* = 15), plus one isolate with no host species information (*n* = 1). Isolates with unspecified origin or host species were submitted by users of our laboratory without accompanying metadata.

### 2.2. Isolation and Microbiological Identification

Bacteriological cultures were performed for bacterial isolation and recovery of frozen stocks. For bacterial isolation, spleen, anterior kidney and brain tissues were aseptically streaked onto Commercial Columbia Blood Agar (CBA) culture plates (Oxoid, Basingstoke, UK) and incubated at 24 °C ± 2 °C in aerobic conditions for 24–48 h. Plates were examined at 24 h and 48 h, and colonies compatible with *Lactococcus* spp. were subcultured to purity on CBA. Pure cultures were stored at −80 °C in cryopreservative containing milk and glycerol until testing.

Preliminary identification of the bacterial isolates was carried out using MALDI-TOF MS (Bruker Daltonics GmbH, Bremen, Germany). As *L. garvieae*, *L. petauri* and *L. formosensis* exhibit highly similar spectra, differentiation at the species level is not possible using the Bruker database, even when the identification score exceeds 2.0. Therefore, isolates yielding a score higher than 1.7 were primarily classified as *Lactococcus* spp. in accordance with the manufacturer’s criteria for probable genus-level identification.

### 2.3. Molecular Identification

Species identification was performed using real-time PCR (qPCR) analysis. DNA was isolated and purified with the MagMax Core extraction kit (Thermo Fisher Scientific, Waltham, MA, USA) using a KingFisher™ Flex Purification System automatic extraction device (Thermo Fisher Scientific, Waltham, MA, USA) following the instructions from the manufacturer.

The qPCR reaction was conducted using commercially available EXOone series kits (Exopol, Zaragoza, Spain) following the user guide. Three different kits were used: “EXOone Lactococcosis causing bacteria” (ref. LGAR), “EXOone Lactococcus garvieae” (ref. XLG1) and “EXOone Lactococcus petauri” (ref. XLG2). The first one is designed for the detection of *L. garvieae*, *L. petauri* and *L. formosensis*, and targets inter-ribosomal region 16S–23S. The other two kits specifically detect *L. garvieae* and *L. petauri*, respectively. For *L. garvieae*, it consists of a duplex qPCR targeting *gly* and *duf* genes. On the other hand, for *L. petauri*, the qPCR targets both *TagG* and *per* genes [35,54]. Additionally, for the strains that could not be identified with the specific qPCR, species identification was achieved through 16S-23S internal transcribed spacer (ITS) region Sanger sequencing externally by Stabvida Laboratories (Caparica, Portugal), following a previously described method [34].

### 2.4. Multilocus Sequence Typing (MLST)

Genetic characterization using multilocus sequence typing (MLST) followed a protocol described previously [37]. The sequences of the conserved regions of seven housekeeping genes (*als*, *atpA*, *tuf*, *gapC*, *gyrB*, *rpoC* and *galP*) were obtained through Sanger sequencing. The obtained sequences were compared with the database in the public repository www.PubMLST.org to determine the corresponding alleles for each of the studied loci.

MLST profiles were analyzed using a goeBURST-like approach to infer clonal complexes (CCs). Allelic profiles for the seven housekeeping loci were retrieved from the public PubMLST database. Pairwise allelic distances were computed as the number of loci with distinct alleles between sequence types (STs). A graph of single-locus variants (SLVs; STs differing at only one locus) was constructed, and connected components of this SLV graph were defined as clonal complexes. Within each CC, the putative founder ST was identified as the one with the highest number of SLVs, with ties resolved by selecting the numerically smallest ST. A minimum spanning tree (MST) was then generated, prioritizing SLV connections and supplemented with double and triple locus variants when necessary to ensure connectivity. STs detected in the study were shown in red circles with white numbers. The resulting diagrams were visualized to highlight the structure of clonal complexes and the relationships among STs.

### 2.5. Antimicrobial Susceptibility Testing

Antimicrobial susceptibility testing was performed using the broth microdilution method to determine the minimum inhibitory concentration (MIC). The test was conducted in Mueller Hinton culture medium (CM0405B-Mueller Hinton Thermo Fisher Scientific, Oxoid™, Basingstoke, UK), supplemented with solution of divalent cations (10 to 12.5 mg of Mg^++^/L (Magnesium chloride, Anhydrous, M8266- Sigma-Aldrich, St. Louis, MO, USA) and 20 to 25 mg of Ca^++^/L (Calcium Chloride anhydrous pure, 141221-PanReac AppliChem, Darmstadt, Germany)) after sterilization [41], and also with 2.5–5% *v*/*v* Laked Horse Blood (SR0048C Thermo Fisher Scientific, Waltham, MA, USA).

MIC plates were prepared with dilutions of the following antimicrobials and the corresponding ranges of final concentrations tested were as follows: oxytetracycline (46598-250MG-Supelco, Bellefonte, PA, USA) 0.063–32 µg/mL, florfenicol (HY-B1374/CS-4857-MedChemExpress, Monmouth Junction, NJ, USA) 0.031–16 µg/mL, flumequine (45735-250MG-Supelco) 0.063–32 µg/mL, amoxicillin (A8523-1G–Sigma-Aldrich, St. Louis, MO, USA) 2–16 µg/mL and trimethoprim–sulfamethoxazole (46984 and 31737-250MG-Supelco, Bellefonte, PA, USA) 0.031–16 µg/mL. Trimethoprim–sulfamethoxazole was used as an indicator of the combination of trimethoprim with another sulfonamide [42].

As internal quality control for each MIC run, reference strains included were *Escherichia coli* ATCC 25922, *Staphylococcus aureus* ATCC 29213 and *Enterococcus faecalis* ATCC 29212 (all from ATCC, Manassas, VA, USA). For agents with CLSI-published MIC quality control ranges for these strains (florfenicol and trimethoprim–sulfamethoxazole) [42], results were required to fall within the listed ranges; any out-of-range run was invalidated and repeated. For agents without ranges published in CLSI VET08 for these quality control strains, we verified assay performance using commercial controls, bibliographic results and/or replicate testing.

To prepare the inoculum, isolated colonies from fresh cultures were suspended in sterile saline solution and adjusted to a turbidity of 0.5 McFarland standard [41]. Once inoculated, the plates were incubated at 35 °C in a 5% CO_2_ atmosphere [55].

The MIC was defined as the lowest antimicrobial concentration that inhibited the bacterial growth. Growth inhibition was determined by comparing the color and turbidity of the wells with the positive and negative control wells on each plate, based on the absence of change. In line with CLSI veterinary standards, in the absence of fish-specific clinical breakpoints, MIC data are presented comparatively (MIC ranges and MIC50/90) because species-specific clinical breakpoints for piscine *Lactococcus* are not established [42].

## 3. Results

### 3.1. Species Identification and Distribution

Of the 39 *Lactococcus* isolates analyzed, all resulted positive in the broad-range EXOone “lactococcosis-causing bacteria” (LCB) qPCR assay, confirming their inclusion within this bacterial group (Cq range: 17–32). Thirty-two isolates were viable and successfully identified as *Lactococcus* spp. by MALDI-TOF MS, yielding scores ≥ 1.7 that supported genus-level identification. Isolates numbered 2, 3, 4, 6, 7, 8, and 9 were non-viable and could not be analyzed by this technique. Species-level discrimination by MALDI-TOF MS was not achievable due to the high spectral similarity among *L. garvieae*, *L. petauri*, and *L. formosensis*.

Species-specific qPCR assays enabled further molecular differentiation: 26 isolates (66.7%) were positive for *L. garvieae* (*gly* and/or *duf* genes) and 10 (25.6%) for *L. petauri* (*TagG* and/or *per* genes), and 3 isolates (7.7%; IDs 26, 32, and 38) yielded negative results for all four species-specific qPCR assays and were therefore initially unclassified. These three isolates were subsequently confirmed as *L. garvieae* through sequencing of the 16S–23S internal transcribed spacer (ITS) region and comparison with sequences deposited in the NCBI GenBank database, showing > 99% homology with *L. garvieae* ATCC 49156 (accession no. AP009332) (Table 2). No isolates were identified as *L. formosensis*.

When grouped by host origin, isolates from marine species yielded only *L. garvieae* (*n* = 20). These comprised European seabass (*Dicentrarchus labrax*; *n* = 15), gilthead seabream (*Sparus aurata*; *n* = 3), and unknown host species (*n* = 2). Among freshwater isolates (*n* = 16), both *L. garvieae* and *L. petauri* were detected (*n* = 8 each). Fifteen isolates were recovered from rainbow trout (*Oncorhynchus mykiss*), with one additional freshwater isolate lacking host information. By period, isolates from 2006 to 2014 were predominantly *L. garvieae* (70%), whereas those from 2021 to 2024 were mostly *L. petauri* (83%).

Among the isolates of unknown origin (*n* = 3), two were classified as *L. petauri* and one as *L. garvieae*.

### 3.2. Genetic Characterization by Multilocus Sequence Typing (MLST)

Multilocus sequence typing (MLST) was successfully performed for all 39 *Lactococcus* isolates, and each isolate was assigned to a sequence type (ST) and corresponding clonal complex (CC) (Table 3).

**Figure 1:**
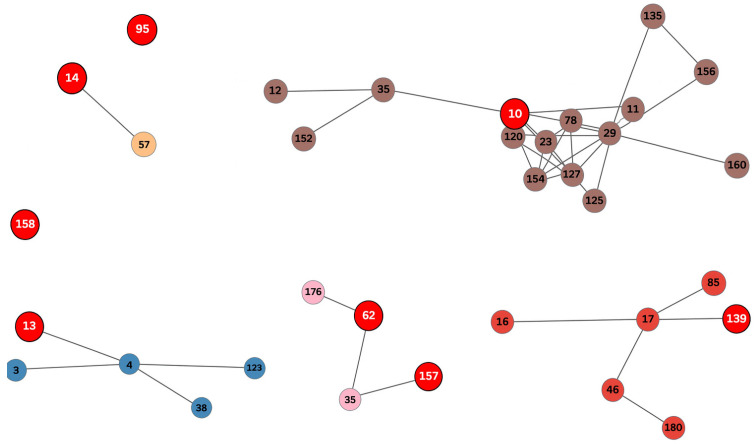
Minimum spanning tree (MST) generated from the seven-locus MLST allelic profiles using a goeBURST-like approach. Each node represents a sequence type (ST), and connections indicate single-locus variants (SLVs). Clonal complexes (CCs) were inferred based on SLV connectivity and putative founder STs were assigned according to the highest number of SLV links. Red circles with white numbers indicate the STs identified in this study, whereas circles with black numbers represent STs retrieved from the www.pubmlst.org database.

The relationships among STs were further examined using a goeBURST-based minimum spanning tree (MST) generated from the allelic profiles, which allowed the visualization of single-locus connections and the distribution of clonal complexes (Figure 1).

The analysis of the seven housekeeping genes revealed the presence of multiple sequence types (STs), ST10, ST13, ST14, ST62, ST95, ST139, ST157 and ST158, all belonging to different clonal complexes (CCs), except for ST62 and ST157 which both were grouped into CC62. Notably, ST157 and ST158 were described for the first time during the development of the present research and were subsequently registered accordingly.

Within the marine isolates, all of which were *L. garvieae*, four different STs were identified. ST95 (CC95) accounted for 80% of the isolates (*n* = 16), being identified both in European seabass and gilthead seabream, as well as in unknown hosts. The following STs were also identified: ST139 (CC17) in 5% (*n* = 1), ST157 (CC62) in 10% (*n* = 2) and ST158 (CC158) in 5% (*n* = 1). All of them came from European seabass.

Among the freshwater isolates, four sequence types (STs) were identified. *L. garvieae* included ST13 (CC4) and ST62 (CC62), whereas *L. petauri* comprised ST10 (CC29) and ST14 (CC14). ST13 and ST14 were the most frequently detected sequence types, each representing 44% (*n* = 7) of the freshwater isolates, while ST10 and ST62 were detected at lower frequencies (*n* = 1 each). The ST62 isolate originated from an unknown host species, whereas all other freshwater isolates were obtained from rainbow trout. Earlier isolates (2006–2014) were predominantly ST13 (70%), with occasional detections of ST14 and ST10. In contrast, recent isolates (2021–2024) were mainly ST14 (83%), with a single ST62 isolate also recorded.

Additionally, two isolates of unknown origin belonged to ST14 (CC14) and one to ST95 (CC95), while CC62 was detected in both European seabass and an unidentified freshwater host.

To facilitate the interpretation of the molecular diversity observed, all *Lactococcus* strains were classified according to species, origin, and host, and grouped by their corresponding sequence type (ST) and clonal complex (CC). The distribution of STs and CCs within each host species, as well as among isolates of unknown origin, is summarized in Table 4.

### 3.3. Antimicrobial Susceptibility Testing by Minimum Inhibition Concentration (MIC)

Antimicrobial susceptibility testing was conducted on 32 viable isolates to determine minimum inhibitory concentration (MIC) values for five commonly used antibiotics (Table 5).

All viable isolates exhibited low MICs for amoxicillin (≤2 µg/mL) across all *L. garvieae* and *L. petauri* tested. In contrast, flumequine showed no inhibitory effect at the highest concentration tested, with MICs > 32 µg/mL for all isolates. For florfenicol, MIC values ranged between 2 and 8 µg/mL. Among *L. garvieae*, all isolates except one (96%), corresponding to strain No. 20, had MICs of 4 µg/mL, whereas in *L. petauri*, seven isolates (78%) shared this same value.

Greater variability was detected for oxytetracycline and trimethoprim–sulfamethoxazole. Oxytetracycline MICs ranged from 0.25 to >32 µg/mL, with 18 *L. garvieae* isolates (78%) showing values between 0.5 and 1 µg/mL, whereas seven *L. petauri* isolates (78%) presented MICs between 1 and 4 µg/mL. One *L. garvieae* isolate (No. 33, ST139) exhibited an MIC > 32 µg/mL.

Trimethoprim–sulfamethoxazole MICs varied from 1 to >16 µg/mL. In *L. garvieae*, fifteen isolates (65%) had MIC values between 8 and >16 µg/mL; in *L. petauri*, eight isolates (89%) fell within the same range.

As shown in Table 6, the minimum inhibitory concentrations required to inhibit 50% (MIC_50_) and 90% (MIC_90_) of the isolates were identical for *L. garvieae* and *L. petauri* with amoxicillin and flumequine. Similar results were observed for florfenicol, whereas oxytetracycline and trimethoprim–sulfamethoxazole displayed greater interspecific variability.

For amoxicillin, both MIC_50_ and MIC_90_ were ≤2 µg/mL in *L. garvieae* and *L. petauri*. For flumequine, MIC_50_ and MIC_90_ were >32 µg/mL for both species, confirming the absence of inhibitory activity at the concentrations tested. In the case of florfenicol, MIC_50_ and MIC_90_ values were nearly identical between species (4 µg/mL), with a possible higher range in *L. petauri* (4–8 µg/mL). Conversely, oxytetracycline and trimethoprim–sulfamethoxazole exhibited higher MIC_50_ and MIC_90_ values in *L. petauri* (4–8 µg/mL and ≥16 µg/mL, respectively) than in *L. garvieae* (1–2 µg/mL and 8–>16 µg/mL, respectively).

## 4. Discussion

This study provides an updated overview of the molecular epidemiology of *L. garvieae* and *L. petauri* based on the combined application of species-specific qPCR assays and multilocus sequence typing (MLST). These complementary approaches allowed the identification and genetic characterization of isolates from both continental and marine origins, contributing to a better understanding of their distribution and evolutionary relationships. All isolates included in the study originated from countries within the Mediterranean region. However, some limitations should be acknowledged. In several cases, the exact geographical origin of the isolates could not be determined, as samples were submitted to the diagnostic laboratory by external partners who, for confidentiality reasons, were unable to release detailed location information. This constraint limited the spatial resolution of the analysis but did not affect the molecular identification or the comparative interpretation of the isolates.

As shown in Table 2 and Table 3, a clear temporal shift can be observed among the freshwater isolates. Those recovered during the 2010s were predominantly identified as *L. garvieae*, with isolates obtained in 2012 and 2013 belonging mainly to ST13 (CC4). In contrast, the freshwater isolates obtained during the 2020s were mostly identified as *L. petauri*. Only one isolate from this decade corresponded to *L. garvieae* and displayed a distinct genetic profile, as it belonged to ST62 (CC62) and tested negative in all qPCR assays targeting species-specific genes. This marked difference suggests the replacement of *L. garvieae* lineages by *L. petauri* over time in continental environments.

Consistently, most isolates identified as *L. petauri* were obtained during the 2020s. The predominant sequence type, ST14 (CC14), although already present in the first decade of the century, has been more frequently detected in the most recent years of the series. This predominance supports the idea of a progressive epidemiological transition in inland waters, where *L. petauri* appears to have largely replaced *L. garvieae*. The *L. petauri* isolates showed a high degree of genetic homogeneity, since all but one belonged to ST14 (CC14). The remaining *L. petauri* isolate differed from this dominant lineage, being characterized as ST10 (CC29) and showing no close genetic relationship with the others.

Within the marine category, all isolates were identified as *L. garvieae*. No temporal trend could be inferred beyond the emergence window, since all marine cases were recorded in 2023 and 2024, which coincides with the first documented outbreaks in European seabass and gilthead seabream aquaculture in Europe [14,15,56]. Most marine isolates (80%; 16/20) belonged to ST95. Remarkably, ST95 was first documented in 2021 in Japan associated with outbreaks in Japanese amberjack (*Seriola quinqueradiata*) [57] and soon after was reported in 2023 in European seabass farms in Italy [14], marking its introduction into European marine aquaculture. Our findings align with this epidemiological pattern, as we also detected ST95 in 2023 in marine seabass samples from Mediterranean aquaculture, supporting the hypothesis that this lineage has recently established itself in the Mediterranean basin. Notably, Japanese isolates belonging to ST95 have been confirmed to represent a novel antigenic variant, designated serotype III [7,57,58,59], which does not cross-react with vaccines designed against serotypes I and II. This observation has direct implications for control strategies, as the emergence of ST95-associated serotype III in the Mediterranean strongly suggests that current vaccine formulations may offer limited protection, reinforcing the need for updated serotype-specific immunoprophylaxis in marine aquaculture systems. Given the growing evidence of geographically expanding ST95/serotype III lineages, vaccine efficacy monitoring and molecular surveillance should be considered priority tools within Mediterranean aquaculture biosecurity programs, as the potential impact on existing vaccines inferred from previous reports underscores the need for region-specific empirical data.

Despite the predominance of ST95, the population was not genetically homogeneous, as ST157 and ST158 were also detected for the first time as well as another single isolate belonging to ST139. The detection of these additional STs, although at lower frequency, suggests that multiple *L. garvieae* lineages are now entering or co-circulating within marine aquaculture systems, potentially reflecting parallel introduction events or diversification following regional establishment.

As summarized in Table 4, these findings highlight the genetic diversity of this microorganism, not only between different production systems and hosts in both continental and marine waters, but also within each of these categories. Such genetic diversity may complicate the accurate identification of *Lactococcus* species. The use of qPCR assays targeting two distinct genes per species enabled the differentiation of most isolates. For *L. petauri*, ST14 isolates were positive for both *tag* and *per* gene assays, whereas the ST10 isolate amplified only the *per* gene. For *L. garvieae*, six sequence types (STs) were detected, showing variable qPCR profiles: ST139 was positive for both *gly* and *duf* targets, ST95 amplified only *gly*, and ST13 and ST158 only *duf*. In contrast, the three *L. garvieae* isolates belonging to ST157 (*n* = 2) and ST62 (*n* = 1) tested negative in both *L. garvieae*-specific qPCR assays and therefore required sequencing of the 16S–23S ITS region for species confirmation.

This study also provides valuable insights by proposing and evaluating the use of qPCR as a rapid and practical diagnostic tool for routine use in diagnostic laboratories. In contrast, sequencing techniques are not yet easily implemented in all laboratories and generally require several days for execution and interpretation. In the vast majority of cases, qPCR successfully enabled species-level identification; however, it failed in three isolates, highlighting a limitation of this approach. Whole-genome sequencing could potentially explain these negative results by revealing polymorphisms or deletions in target genes [60], but was unavailable during this study. Species identification therefore relied on routinely used diagnostic methods such as ITS Sanger sequencing [34]. Despite this limitation, all isolates were reliably identified, and the study objectives were successfully achieved.

For three isolates, the aquatic environment of origin could not be determined, as they were submitted without complete metadata and were therefore classified as of unknown origin (freshwater or marine). However, their molecular identification and sequence typing results allow reasonable inferences about their likely provenance. The two isolates identified as *L. petauri* and assigned to ST14 (CC14) shared identical molecular profiles with other continental isolates of the same sequence type, suggesting that they most probably originated from freshwater aquaculture systems and were likely isolated from rainbow trout. In contrast, the *L. garvieae* isolate of unknown origin, classified as ST95 (CC95), showed full concordance with the genetic characteristics of the marine isolates included in this study, indicating a likely marine origin and probably a host species like European seabass or gilthead seabream.

No coinfections between *L. garvieae* and *L. petauri* could be assessed in this study, as all samples included in the dataset corresponded to pure cultures obtained after microbiological isolation. Consequently, the study design does not allow the evaluation of potential mixed infections at the tissue level, which should be regarded as a limitation of the present work. Nevertheless, the molecular approach proposed in this study could, in future applications, be used directly on fish tissues, potentially enabling the detection of mixed infections and overcoming some of the limitations inherent to culture-based diagnostics. In this context, qPCR represents a promising tool for rapid and species-specific detection in epidemiological and diagnostic settings, although this application was not evaluated within the scope of the present study. Additionally, no cases of *L. formosensis* were detected, which is epidemiologically relevant considering recent reports distinguishing this species from *L. garvieae* serotype II in marine outbreaks in Asia [28,59] and also its recent first description in rainbow trout in Europe [32]. The absence of *L. formosensis* in our Mediterranean samples reinforces the consistency of the regional epidemiological profile, currently dominated by *L. garvieae* ST95 in marine systems and *L. petauri* ST14 in continental aquaculture.

In this study, we analyzed information available from the PUBMLST database (www.pubmlst.org, accessed in October 2025) regarding the isolates corresponding to the sequence types (STs) identified in our work, as well as those closely related (single-locus variants, SLVs) within their respective clonal complexes (CCs). A summary of the included isolates is available in the Supplementary Materials (Table S2). This approach allowed us to contextualize our findings, exploring temporal, geographic and host diversity, and to discuss their potential zoonotic implications.

Within CC14, ST14 emerged as a persistent aquaculture-associated lineage, detected in our study from 2006 to 2024 and consistently recovered from *Oncorhynchus mykiss* farmed in continental freshwater systems. All isolates belonging to this ST were identified as *L. petauri*, with no evidence of host or ecological diversification within our dataset. These findings mirror the epidemiological pattern reported in PubMLST, where ST14 is largely confined to intensive freshwater aquaculture in Mediterranean countries, particularly Spain and Turkey. Notably, ST57, a single-locus variant of ST14, was not detected in our study; however, PubMLST records include a single human clinical case reported in Spain in 2013. Its *L. petauri* designation suggests an origin from the aquaculture-associated ST14 lineage, representing a rare, opportunistic spillover rather than a human-adapted strain.

CC62, which includes ST62 and ST157 (both found in our work) and the closely related ST86, shows a distribution spanning both the Mediterranean region and more distant locations (India). Isolates were recovered from different hosts, cattle and fish, all consistently identified as *L. garvieae*. ST62 is particularly noteworthy for its ecological plasticity, being detected in bovine mastitis cases in Spain (2000) and in rainbow trout in India (2015), suggesting both spillover between livestock and fish and intercontinental spread. In our work, we detected ST62 in freshwater species while ST157 was detected in marine species (described for the first time here). ST86 has been exclusively isolated from cattle. Taken together, this cluster appears to be primarily associated with livestock and aquaculture, both marine and continental; however, its potential to circulate between these systems has yet to be conclusively demonstrated. ST139, considered together with the closely related ST17 (both belonging to CC17), is mainly found in Spain. ST17 PubMLST records concentrated between 2009 and 2011, while ST139 was first detected by our laboratory (unpublished) in another strain from a marine fish species in the year 2023. The predominant host for CC17 is fish, although human isolates have also been reported, all identified as *L. garvieae*. This pattern is compatible with a zoonotic character, in which a fish-associated lineage can colonize or infect humans through handling or consumption of fish products. CC17 was also previously described in marine species in Japan and South Korea and tilapia in Brazil.

CC29, encompassing ST10 (detected once in this study) and several SLVs such as ST29, ST28, ST23, ST154 and ST11, represents one of the largest clusters and is present in both Europe (Spain and Greece) and South America. Host distribution is mixed, including fish, humans and cattle. The exclusive species recorded is *L. petauri*, consistent with our findings. The coexistence of human and fish isolates over time demonstrates sustained interspecies transmission, with clear zoonotic implications and a potential role in bridging aquaculture and public health.

CC4, comprising ST3, ST4, ST13, ST38 and ST123, spans the longest temporal range (1981–2024) and displays a broad geographic distribution, with Italy as the main contributor, followed by the USA, Brazil, Spain and Malaysia. This cluster shows the greatest host diversity, including isolates from fish, cattle, milk destined for human consumption and humans. *L. garvieae* is the only species, in line with our findings, and is well recognized for its zoonotic potential. The wide spectrum of hosts and sources positions this complex as an ecological bridge connecting aquaculture, livestock and human health, emphasizing the need for its inclusion in the surveillance of emerging zoonoses.

Taken together, the analysis of these clonal complexes reveals multiple examples of spillover between hosts, particularly between fish and cattle (CC62) and between fish and humans (CC17, CC29 and CC4). The circulation of these lineages across diverse environments and hosts, together with their temporal persistence and wide geographic distribution, reinforces the view of *L. garvieae* and *L. petauri* as bacteria posing a genuine zoonotic risk at the aquaculture–livestock–human interface.

The antimicrobial susceptibility results provide a comparative overview of current MIC distributions in *L. garvieae* and *L. petauri*. Because fish-specific clinical breakpoints for *Lactococcus* spp. are not established, we interpret our data as MIC ranges and MIC_50_/MIC_90_ values rather than categorical susceptible/intermediate/resistance (S/I/R) calls, in line with CLSI veterinary antimicrobial susceptibility testing guidance [41,42]. We complement these results with recent epidemiological cutoff value (ECV) data reported for *L. garvieae* and *L. petauri* [61].

Overall, MIC values for amoxicillin, flumequine, and florfenicol showed little variability, both within and between species. In contrast, oxytetracycline and trimethoprim–sulfamethoxazole displayed greater interspecific variation, suggesting potential differences in resistance mechanisms or selective pressures between the two species. It should be noted that seven older isolates could not be included in MIC testing due to loss of viability after long-term storage, slightly limiting comparisons between historical freshwater and more recent marine *L. garvieae* isolates.

Amoxicillin showed uniformly low MIC values (≤2 µg/mL) across all isolates, consistent with multi-country datasets in which *L. garvieae* and *L. petauri* display low ECVs for β-lactams [61]. This class of antimicrobials could potentially therefore serve as a first-line treatment for lactococcosis in fish, as suggested by the aquaculture sector [49].

Florfenicol MICs clustered between 2 and 8 µg/mL, with the majority at 4 µg/mL, indicating a narrow distribution and suggesting that acquired resistance remains uncommon in our Mediterranean samples. This pattern aligns with a recent North American isolate study [30], which also found a narrow distribution of MIC values, and another Japanese susceptibility study for *L. garvieae* [7], which found equivalent values to the ones seen in this study.

In contrast, flumequine showed no inhibitory activity at the concentrations tested (MIC > 32 µg/mL) for either species, reflecting its limited activity against Gram-positive bacteria [47]. This is consistent with field observations where fluoroquinolone treatments have shown limited effectiveness during *L. garvieae* outbreaks in marine fish (reported as treatment failure or poor response), though those reports often used disk diffusion rather than MIC; therefore, we refrain from direct method comparisons [14]. Collectively, our MIC findings and recent case data do not support routine use of flumequine against lactococcosis. Coltraro et al. [56] recently reported the first documented outbreaks of *L. garvieae* in gilthead seabream from the Gulf of Follonica (Tuscany, Italy), providing a direct regional comparison of antimicrobial susceptibility. Our MIC results closely match theirs for amoxicillin, florfenicol, oxytetracycline and flumequine, confirming similar susceptibility patterns. The only minor difference appears in trimethoprim–sulfamethoxazole, where our MIC_50_ value (8 µg/mL) is slightly higher than their reported 2 µg/mL, likely due to clonal variation or small methodological differences. For oxytetracycline, MIC_50_ and MIC_90_ values ranged from 1 to 2 µg/mL in *L. garvieae* to 4–8 µg/mL in *L. petauri*. This variation may reflect species-specific differences in intrinsic susceptibility or selective pressure from tetracycline use in freshwater trout farms. Notably, one marine isolate (strain No. 33, ST139) displayed a markedly elevated MIC (>32 µg/mL), representing a potential case of acquired resistance that warrants further molecular investigation. This variability in MIC values between isolates was also found in a recent report [61].

Trimethoprim–sulfamethoxazole exhibited generally elevated MICs (MIC_90_ > 16 µg/mL), consistent with recent reports indicating high ECVs for both species in the Mediterranean region [61].

Due to the rapid mortality of *L. garvieae* infections requiring immediate intervention during commercial outbreaks [5,14], studies like ours provide essential epidemiological data by updating antimicrobial resistance profiles of strains from recent Mediterranean outbreaks. Antimicrobial susceptibility testing timelines (≥48 h) limit routine use, supporting empirical therapy with authorized agents such as erythromycin, oxytetracycline, and florfenicol [62], while prioritizing biosecurity and vaccination. Limitations include the absence of fish-specific breakpoints and variable marine pharmacokinetics, underscoring the value of region-specific surveillance.

Overall, these results indicate that the uses of amoxicillin, oxytetracycline and florfenicol appear to be viable first-line empirical options while waiting, as rapidly as possible, for laboratory antimicrobial resistance confirmation from antimicrobial susceptibility testing. The consistently high MICs observed for flumequine and trimethoprim–sulfamethoxazole suggest that they should not be considered as first options for treating *Lactococcus* infections in fish. These findings highlight the importance of regular MIC monitoring to ensure responsible antimicrobial use and maintain therapeutic efficacy.

The data presented in this study point to a complex and evolving epidemiology of lactococcosis and suggest a potential for *L. garvieae* and *L. petauri* lineages to cross ecological and host barriers. Further studies specifically designed to characterize the capacity of different lineages to cross species boundaries would be of particular interest. Their zoonotic potential and ability to acquire antimicrobial resistance emphasize the need for monitoring and control measures that integrate animal, environmental and human health perspectives. Although this work focused on genetic diversity, antigenic variation is expected to accompany genomic divergence, reinforcing the importance of developing effective immunization strategies, including tailored autovaccines adapted to each production unit. Overall, the findings reinforce the value of adopting a One Health framework to manage *Lactococcus* infections, integrating molecular surveillance, antimicrobial susceptibility testing and serotype monitoring within coordinated health programs. The baseline data generated here contribute to both aquaculture disease prevention and public health preparedness, underscoring that protecting fish health is an essential component of global health security.

## 5. Conclusions

We successfully traced the genetic and antimicrobial profiles of *L. garvieae* and *L. petauri* circulating in Mediterranean aquaculture. Our data reveal a shift from *L. garvieae* ST13 to *L. petauri* ST14 as the dominant freshwater lineage, while marine outbreaks remain linked to *L. garvieae* ST95. Amoxicillin showed low MIC values, whereas oxytetracycline and trimethoprim–sulfamethoxazole showed variable results. Continuous region-specific molecular and AMR data surveillance provides updated epidemiological profiles as an immediate actionable tool for managing emerging *Lactococcus* lineages and safeguarding Mediterranean aquaculture productivity.

## Figures and Tables

**Table 1:** Number of strains for each origin and host species.

Origin	Host Species	*n*
Marine	*Dicentrarchus labrax*	15
	*Sparus aurata*	3
	Not specified	2
Freshwater	*Oncorhynchus mykiss*	15
	Not specified	1
Unspecified	Not specified	3

**Table 2:** Molecular identification results for the 39 *Lactococcus* strains with the corresponding number, year of isolation, origin and host species.

Strain	Year	Origin	Host	LCB ^1^	LGAR *gly* ^2^	LGAR *duf* ^3^	LPET *tag* ^4^	LPET *per* ^5^	Identification
				qPCR (Cq Value)					
1	2006	freshwater	*Oncorhynchus mykiss*	23	neg *	neg	25	25	*L. petauri*
2	2012	freshwater	*Oncorhynchus mykiss*	20	neg	neg	22	22	*L. petauri*
3	2012	freshwater	*Oncorhynchus mykiss*	24	neg	27	neg	neg	*L. garvieae*
4	2013	freshwater	*Oncorhynchus mykiss*	23	neg	23	neg	neg	*L. garvieae*
5	2013	freshwater	*Oncorhynchus mykiss*	24	neg	25	neg	neg	*L. garvieae*
6	2013	freshwater	*Oncorhynchus mykiss*	21	neg	23	neg	neg	*L. garvieae*
7	2013	freshwater	*Oncorhynchus mykiss*	21	neg	24	neg	neg	*L. garvieae*
8	2013	freshwater	*Oncorhynchus mykiss*	19	neg	22	neg	neg	*L. garvieae*
9	2013	freshwater	*Oncorhynchus mykiss*	20	neg	22	neg	neg	*L. garvieae*
10	2014	freshwater	*Oncorhynchus mykiss*	24	neg	neg	neg	26	*L. petauri*
11	2021	freshwater	*Oncorhynchus mykiss*	25	neg	neg	27	27	*L. petauri*
12	2022	freshwater	*Oncorhynchus mykiss*	19	neg	neg	22	22	*L. petauri*
13	2022	freshwater	*Oncorhynchus mykiss*	22	neg	neg	26	25	*L. petauri*
14	2022	freshwater	*Oncorhynchus mykiss*	25	neg	neg	27	26	*L. petauri*
15	2023	marine	*Dicentrarchus labrax*	22	25	neg	neg	neg	*L. garvieae*
16	2023	marine	*Dicentrarchus labrax*	17	20	neg	neg	neg	*L. garvieae*
17	2023	marine	*Dicentrarchus labrax*	24	26	neg	neg	neg	*L. garvieae*
18	2023	marine	*Dicentrarchus labrax*	24	25	neg	neg	neg	*L. garvieae*
19	2023	marine	*Sparus aurata*	24	26	neg	neg	neg	*L. garvieae*
20	2023	marine	*Dicentrarchus labrax*	22	24	neg	neg	neg	*L. garvieae*
21	2023	marine	Unknown	25	27	neg	neg	neg	*L. garvieae*
22	2024	unknown	Unknown	24	neg	neg	25	25	*L. petauri*
23	2024	marine	*Dicentrarchus labrax*	26	27	neg	neg	neg	*L. garvieae*
24	2024	unknown	Unknown	22	neg	neg	22	24	*L. petauri*
25	2024	marine	*Dicentrarchus labrax*	23	23	neg	neg	neg	*L. garvieae*
26	2024	freshwater	Unknown	32	neg	neg	neg	neg	*L. garvieae*
27	2024	unknown	Unknown	26	26	neg	neg	neg	*L. garvieae*
28	2024	marine	Unknown	26	29	neg	neg	neg	*L. garvieae*
29	2024	freshwater	*Oncorhynchus mykiss*	24	neg	neg	26	26	*L. petauri*
30	2024	marine	*Dicentrarchus labrax*	25	26	neg	neg	neg	*L. garvieae*
31	2024	marine	*Dicentrarchus labrax*	21	neg	24	neg	neg	*L. garvieae*
32	2024	marine	*Dicentrarchus labrax*	25	neg	neg	neg	neg	*L. garvieae*
33	2024	marine	*Dicentrarchus labrax*	24	25	26	neg	neg	*L. garvieae*
34	2024	marine	*Sparus aurata*	25	26	neg	neg	neg	*L. garvieae*
35	2024	marine	*Sparus aurata*	25	27	neg	neg	neg	*L. garvieae*
36	2024	marine	*Dicentrarchus labrax*	26	27	neg	neg	neg	*L. garvieae*
37	2024	marine	*Dicentrarchus labrax*	25	26	neg	neg	neg	*L. garvieae*
38	2024	marine	*Dicentrarchus labrax*	27	neg	neg	neg	neg	*L. garvieae*
39	2024	marine	*Dicentrarchus labrax*	26	26	neg	neg	neg	*L. garvieae*

^1^ LCB = qPCR for Lactococcosis-causing bacteria; ^2^ LGAR *gly* = qPCR for *L. garvieae*, *gly* gene; ^3^ LGAR *duf* = qPCR for *L. garvieae*, *duf* gene; ^4^ LPET *tag* = qPCR for *L. petauri*, *tag* gene; ^5^ LPET *per* = qPCR for *L. petauri*, *per* gene; * neg = negative.

**Table 3:** Genetic characterization of *Lactococcus* isolates by multilocus sequence typing (MLST), showing allelic profiles for each gene, sequence type (ST) and clonal complex (CC) assignments.

Strain	Year	Host	Identification	*als*	*atpA*	*tuf*	*gapC*	*gyrB*	*rpoC*	*galP*	ST	CC
1	2006	*Oncorhynchus mykiss*	*L. petauri*	11	6	10	2	9	11	8	14	CC14
2	2012	*Oncorhynchus mykiss*	*L. petauri*	11	6	10	2	9	11	8	14	CC14
3	2012	*Oncorhynchus mykiss*	*L. garvieae*	10	3	4	2	3	3	3	13	CC4
4	2013	*Oncorhynchus mykiss*	*L. garvieae*	10	3	4	2	3	3	3	13	CC4
5	2013	*Oncorhynchus mykiss*	*L. garvieae*	10	3	4	2	3	3	3	13	CC4
6	2013	*Oncorhynchus mykiss*	*L. garvieae*	10	3	4	2	3	3	3	13	CC4
7	2013	*Oncorhynchus mykiss*	*L. garvieae*	10	3	4	2	3	3	3	13	CC4
8	2013	*Oncorhynchus mykiss*	*L. garvieae*	10	3	4	2	3	3	3	13	CC4
9	2013	*Oncorhynchus mykiss*	*L. garvieae*	10	3	4	2	3	3	3	13	CC4
10	2014	*Oncorhynchus mykiss*	*L. petauri*	9	7	3	4	7	9	9	10	CC29
11	2021	*Oncorhynchus mykiss*	*L. petauri*	11	6	10	2	9	11	8	14	CC14
12	2022	*Oncorhynchus mykiss*	*L. petauri*	11	6	10	2	9	11	8	14	CC14
13	2022	*Oncorhynchus mykiss*	*L. petauri*	11	6	10	2	9	11	8	14	CC14
14	2022	*Oncorhynchus mykiss*	*L. petauri*	11	6	10	2	9	11	8	14	CC14
15	2023	*Dicentrarchus labrax*	*L. garvieae*	52	38	6	24	45	40	44	95	CC95
16	2023	*Dicentrarchus labrax*	*L. garvieae*	52	38	6	24	45	40	44	95	CC95
17	2023	*Dicentrarchus labrax*	*L. garvieae*	52	38	6	24	45	40	44	95	CC95
18	2023	*Dicentrarchus labrax*	*L. garvieae*	52	38	6	24	45	40	44	95	CC95
19	2023	*Sparus aurata*	*L. garvieae*	52	38	6	24	45	40	44	95	CC95
20	2023	*Dicentrarchus labrax*	*L. garvieae*	52	38	6	24	45	40	44	95	CC95
21	2023	Unknown	*L. garvieae*	52	38	6	24	45	40	44	95	CC95
22	2024	Unknown	*L. petauri*	11	6	10	2	9	11	8	14	CC14
23	2024	*Dicentrarchus labrax*	*L. garvieae*	52	38	6	24	45	40	44	95	CC95
24	2024	Unknown	*L. petauri*	11	6	10	2	9	11	8	14	CC14
25	2024	*Dicentrarchus labrax*	*L. garvieae*	52	38	6	24	45	40	44	95	CC95
26	2024	Unknown	*L. garvieae*	34	24	27	15	28	27	29	62	CC62
27	2024	Unknown	*L. garvieae*	52	38	44	24	45	40	6	95	CC95
28	2024	Unknown	*L. garvieae*	52	38	6	24	45	40	44	95	CC95
29	2024	*Oncorhynchus mykiss*	*L. petauri*	11	6	10	2	9	11	8	14	CC14
30	2024	*Dicentrarchus labrax*	*L. garvieae*	52	38	6	24	45	40	44	95	CC95
31	2024	*Dicentrarchus labrax*	*L. garvieae*	97	66	58	2	5	27	27	158	CC158
32	2024	*Dicentrarchus labrax*	*L. garvieae*	34	24	27	39	42	27	29	157	CC62
33	2024	*Dicentrarchus labrax*	*L. garvieae*	79	8	6	7	10	13	12	139	CC17
34	2024	*Sparus aurata*	*L. garvieae*	52	38	6	24	45	40	44	95	CC95
35	2024	*Sparus aurata*	*L. garvieae*	52	38	6	24	45	40	44	95	CC95
36	2024	*Dicentrarchus labrax*	*L. garvieae*	52	38	6	24	45	40	44	95	CC95
37	2024	*Dicentrarchus labrax*	*L. garvieae*	52	38	44	24	45	40	6	95	CC95
38	2024	*Dicentrarchus labrax*	*L. garvieae*	34	24	27	39	42	27	29	157	CC62
39	2024	*Dicentrarchus labrax*	*L. garvieae*	52	38	44	24	45	40	6	95	CC95

**Table 4:** Summary of ST and CC results for strains of each origin and host species.

*Lactococcus* sp.	Origin	Host	ST	CC	*n*
*L. garvieae*	marine	*Dicentrarchus labrax*	95	95	11
			157	62	2
			139	17	1
			158	158	1
		*Sparus aurata*	95	95	3
		unknown	95	95	2
	freshwater	*Oncorhynchus mykiss*	13	4	7
		unknown	62	62	1
	unknown	unknown	95	95	1
*L. petauri*	freshwater	*Oncorhynchus mykiss*	14	14	7
			10	29	1
	unknown	unknown	14	14	2

**Table 5:** MIC results of amoxicillin, oxytetracycline, trimethoprim–sulfamethoxazole, florfenicol and flumequine for viable strains.

Strain	Year	Host	Identification	ST	CC	MIC AMX ^1^ (µg/mL)	MIC OTC ^2^ (µg/mL)	MIC SXT ^3^ (µg/mL)	MIC FFC ^4^ (µg/mL)	MIC FLU ^5^ (µg/mL)
1	2006	*Oncorhynchus mykiss*	*L. petauri*	14	CC14	≤2	4	>16	4	>32
5	2013	*Oncorhynchus mykiss*	*L. garvieae*	13	CC4	≤2	4	8	4	>32
10	2014	*Oncorhynchus mykiss*	*L. petauri*	10	CC29	≤2	4	16	4	>32
11	2021	*Oncorhynchus mykiss*	*L. petauri*	14	CC14	≤2	2	8	4	>32
12	2022	*Oncorhynchus mykiss*	*L. petauri*	14	CC14	≤2	2	>16	8	>32
13	2022	*Oncorhynchus mykiss*	*L. petauri*	14	CC14	≤2	4	>16	4	>32
14	2022	*Oncorhynchus mykiss*	*L. petauri*	14	CC14	≤2	8	8	2	>32
15	2023	*Dicentrarchus labrax*	*L. garvieae*	95	CC95	≤2	1	2	4	>32
16	2023	*Dicentrarchus labrax*	*L. garvieae*	95	CC95	≤2	1	2	4	>32
17	2023	*Dicentrarchus labrax*	*L. garvieae*	95	CC95	≤2	1	8	4	>32
18	2023	*Dicentrarchus labrax*	*L. garvieae*	95	CC95	≤2	1	>16	4	>32
19	2023	*Sparus aurata*	*L. garvieae*	95	CC95	≤2	1	>16	4	>32
20	2023	*Dicentrarchus labrax*	*L. garvieae*	95	CC95	≤2	2	>16	8	>32
21	2023	Unknown	*L. garvieae*	95	CC95	≤2	1	>16	4	>32
22	2024	Unknown	*L. petauri*	14	CC14	≤2	4	16	4	>32
23	2024	*Dicentrarchus labrax*	*L. garvieae*	95	CC95	≤2	1	4	4	>32
24	2024	Unknown	*L. petauri*	14	CC14	≤2	8	>16	4	>32
25	2024	*Dicentrarchus labrax*	*L. garvieae*	95	CC95	≤2	1	16	4	>32
26	2024	Unknown	*L. garvieae*	62	CC62	≤2	0.25	2	4	>32
27	2024	Unknown	*L. garvieae*	95	CC95	≤2	0.5	8	4	>32
28	2024	Unknown	*L. garvieae*	95	CC95	≤2	0.5	8	4	>32
29	2024	*Oncorhynchus mykiss*	*L. petauri*	14	CC14	≤2	1	1	4	>32
30	2024	*Dicentrarchus labrax*	*L. garvieae*	95	CC95	≤2	0.5	8	4	>32
31	2024	*Dicentrarchus labrax*	*L. garvieae*	158	CC158	≤2	1	2	4	>32
32	2024	*Dicentrarchus labrax*	*L. garvieae*	157	CC62	≤2	0.5	4	4	>32
33	2024	*Dicentrarchus labrax*	*L. garvieae*	139	CC17	≤2	>32	>16	4	>32
34	2024	*Sparus aurata*	*L. garvieae*	95	CC95	≤2	1	8	4	>32
35	2024	*Sparus aurata*	*L. garvieae*	95	CC95	≤2	1	2	4	>32
36	2024	*Dicentrarchus labrax*	*L. garvieae*	95	CC95	≤2	2	16	4	>32
37	2024	*Dicentrarchus labrax*	*L. garvieae*	95	CC95	≤2	0.5	8	4	>32
38	2024	*Dicentrarchus labrax*	*L. garvieae*	157	CC62	≤2	0.5	4	4	>32
39	2024	*Dicentrarchus labrax*	*L. garvieae*	95	CC95	≤2	1	16	4	>32

^1^ AMX = amoxicillin; ^2^ OTC = oxytetracycline; ^3^ SXT = trimethoprim–sulfamethoxazole; ^4^ FFC = florfenicol; ^5^ FLU = flumequine.

**Table 6:** MIC_50_ and MIC_90_ results for *L. garvieae* and *L. petauri*.

	*L. garvieae* (*n* = 23)		*L. petauri* (*n* = 9)	
	MIC_50_ (µg/mL)	MIC_90_ (µg/mL)	MIC_50_ (µg/mL)	MIC_90_ (µg/mL)
Amoxicillin	≤2	≤2	≤2	≤2
Oxytetracycline	1	2	4	8
Trimethoprim–sulfametoxazole	8	>16	16	>16
Florfenicol	4	4	4	4–8 *
Flumequine	>32	>32	>32	>32

* Due to the limited number of isolates and the distribution of MIC values, the exact MIC_90_ concentration could not be precisely determined, although it is estimated to lie within this range.

## Data Availability

The original contributions presented in this study are included in the Supplementary Materials. Further inquiries can be directed to the corresponding authors. Data included in Supplementary Material Table S2 was derived from the resource available in the public domain: Public databases for molecular typing and microbial genome diversity (www.pubmlst.org).

## Supplementary Materials

The following supporting information can be downloaded at https://www.mdpi.com/article/10.3390/ani16020277/s1: Table S1: Complete results for the isolates studied; Table S2: Summary of PUBMLST database regarding the isolates corresponding to the sequence types (STs) identified in the study and single-locus variants (SLVs) within their respective clonal complexes (CCs).

## Abbreviations

The following abbreviations are used in this manuscript:

CC	Clonal complex
CBA	Columbia blood agar
DNA	Deoxyribonucleic acid
ECV	Epidemiological cutoff value
ITS	Internal transcribed spacer
LCB	Lactococcosis causing bacteria
MALDI-TOF MS	Matrix-assisted laser desorption ionization–time-of-flight mass spectrometry
MIC	Minimum inhibitory concentration
MIC_50_	Concentration of an antibiotic required to inhibit the growth of 50% of the strains analyzed
MIC_90_	Concentration of an antibiotic required to inhibit the growth of 90% of the strains analyzed
MLST	Multilocus sequence typing
MST	Minimum spanning tree
PCR	Polymerase chain reaction
qPCR	Real-time PCR
SLV	Single-locus variant
ST	Sequence type
